# Serotonin transporter binding of [^123^I]ADAM in bulimic women, their healthy twin sisters, and healthy women: a SPET study

**DOI:** 10.1186/1471-244X-7-19

**Published:** 2007-05-21

**Authors:** Anu K Koskela, Anna Keski-Rahkonen, Elina Sihvola, Tomi Kauppinen, Jaakko Kaprio, Aapo Ahonen, Aila Rissanen

**Affiliations:** 1 Department of Clinical Physiology and Nuclear Medicine, HUSLAB/Helsinki University Central Hospital, PL 340, 00029 HUS, Helsinki, Finland; 2 Department of Public Health, Helsinki University, Helsinki, Finland; 3 HUS Helsinki Medical Imaging Center, Helsinki University Central Hospital, Helsinki, Finland; 4 Department of Psychiatry, Helsinki University Central Hospital, Helsinki, Finland; 5 Department of Mental Health and Alcohol Research, National Public Health Institute, Helsinki, Finland

## Abstract

**Background:**

Bulimia Nervosa (BN) is believed to be caused by an interaction of genetic and environmental factors. Previous studies support the existence of a bulimia-related endophenotype as well as disturbances in serotonin (5-HT) transmission. We studied serotonin transporter (SERT) binding in BN, and to investigate the possibility of a SERT-related endophenotype for BN, did this in a sample of female twins. We hypothesized clearly reduced SERT binding in BN women as opposed to healthy women, and intermediate SERT binding in unaffected co-twins.

**Methods:**

We studied 13 female twins with BN (9 with purging and 4 with non-purging BN) and 25 healthy women, including 6 healthy twin sisters of BN patients and 19 women from 10 healthy twin pairs. [^123^I]ADAM, a selective SERT radioligand for single photon emission tomography (SPET) imaging, was used to assess SERT availability in the midbrain and the thalamus.

**Results:**

No differences in SERT binding were evident when comparing the BN women, their unaffected co-twins and the healthy controls (p = 0.14). The healthy sisters of the BN patients and the healthy control women had similar SERT binding in both brain regions. In a *post hoc *subgroup analysis, the purging bulimics had higher SERT binding than the healthy women in the midbrain (p = 0.03), but not in the thalamus.

**Conclusion:**

Our finding of increased SERT binding in the midbrain in the purging BN women raises the possibility that this subgroup of bulimics might differ in serotonergic function from the non-purging ones. The similarity of the unaffected co-twins and the healthy controls doesn't support our initial assumption of a SERT-related endophenotype for BN. Due to the small sample size, our results need to be interpreted with caution and verified in a larger sample.

## Background

Bulimia Nervosa (BN) is a common eating disorder that predominantly affects young women. It is characterized by body image distortions and episodes of excessive eating, followed by compensatory behaviors, such as strict dieting, vomiting, laxative misuse or compulsive exercise [1]. BN is often accompanied by disturbances of mood and impulse control [2].

The neurotransmitter serotonin (5-HT), involved in the regulation of eating behavior [3], may play a role in the pathophysiology of BN. Subjects with symptomatic BN show several abnormalities in 5-HT metabolism and function [4-9]. Both women with current BN and women who have recovered from BN seem more vulnerable than healthy control women to the mood lowering and the binge precipitating effects of acute tryptophan depletion [10,11]. A wide range of antidepressant medications are effective in reducing binge and purge frequency [12,13].

It is uncertain whether the changes in 5-HT system in BN are trait dependent (i.e., representing a predisposition to an illness) or state dependent (i.e., resulting from changes in nutritional status during an acute illness). There is some evidence suggesting a bulimia-related endophenotype, such as premorbid anxiety symptoms [14] as well as serotonergic alterations that remain after recovery [11,15,16]. Evidence from family and twin studies suggests a genetic contribution to the etiology of eating disorders [17], but consistent evidence of genetic variation specifically affecting serotonin metabolism disturbances in BN is lacking. Twin samples offer an opportunity to explore state and trait related differences, given that the healthy twins share either all or half their segregating genes with their affected co-twin, depending on whether the twin pair is monozygotic (MZ) or dizygotic (DZ). Moreover, twins are of the same age and have generally similar childhood and adolescent experiences. Observed differences between women with BN and their sisters are likely due to state effects, whereas differences observed between healthy twin sisters and unrelated controls would be trait-related.

The recent developments of new radioligands for various neurotransmitter systems for positron emission tomography (PET) and single photon emission tomography (SPET) imaging have enabled more direct, *in vivo *studies of neurotransmitter dysfunction in different conditions. Previous studies have suggested reduced availability of brain serotonin transporters (SERTs) in BN [18] and Binge Eating Disorder (BED) [19]. There are reports on increased 5-HT_1A_-binding in BN [20] as well as on reduced 5-HT_2A_-binding in women who have recovered from BN [16] and from bulimia-type Anorexia Nervosa [21].

Iodine 123-labeled 2-((2-((dimethylamino)methyl)phenyl)thio)-5-iodophenylamine ([^123^I]ADAM) is a selective radioligand for SPET imaging of SERTs. Its affinity to SERTs is more than 1000-fold over its affinity to dopamine transporters and norepinephrine transporters [22]. Its binding is greatest [23,24] and least variable in a test-retest setting in the midbrain and the thalamus [23], and reaches a pseudoequilibrium at 4-6 h post injection [23]. Its effective dose is similar to the other commonly used radioligands [25,26]. We have previously created an [^123^I]ADAM brain template and a predefined VOI (volume of interest) map for the automated registration and analysis of the [^123^I]ADAM images [27].

The aim of this study was to (1) investigate whether the finding of reduced SERT binding in subjects with BN observed using the less selective radioligand [^123^I]β-CIT [18] can be replicated with [^123^I]ADAM, and (2) to explore the possible genetic background of BN by comparing [^123^I]ADAM binding to SERTs between subjects with BN, their healthy co-twins and healthy controls. We assumed that SERT transmission is a bulimia-related endophenotype, i.e., a heritable quantitative trait that is state-independent (manifest in the individual whether or not illness is active), and found more often in unaffected family members than in the general population. Therefore, we hypothesized that SERT availability would be clearly reduced in SERT rich brain areas in BN women compared to unaffected women, and that the SERT availability of unaffected co-twins would be intermediate between that of probands and healthy women.

## Methods

### Study participants

The study participants were recruited from FinnTwin16, a population-based, longitudinal study including virtually all Finnish twins born 1975-1979 and first studied at the age of 16 [28]. At the age of 22-27 years, the 2545 female twins were screened for eating disorders by self-report questionnaire: 292 screen-positive women, their 130 female co-twins, and 210 screen-negative women were then interviewed using a short version of the SCID-I interview [29] (interview participation rate 85.2%) [30]. From the interviews, we obtained lifetime diagnoses of anorexia, bulimia, binge eating disorder, major depression (MDD) and obsessive-compulsive disorder (OCD). All women suffering from BN who were not on current serotonergic medication (n = 13) as well as their female co-twins were invited to participate in the [^123^I]ADAM study. The DSM-IV diagnosis of bulimia nervosa of each case detected was further confirmed with semi-structured EATATE [31] and SSAGA interviews [32,33]. We chose to also include two women recently recovered from BN in this group on the basis of the evidence suggesting a trait for BN.

Ten healthy, medication-free control female twin pairs without bulimic symptoms were selected from the same FinnTwin16 cohort and invited to participate in the [^123^I]ADAM study. They were also assessed with EATATE and SSAGA interviews, which ruled out past or present eating disorders and revealed one case with a former history of depression. We thus excluded her from our study. Another healthy control was excluded due to an unsuccessful [^123^I]ADAM injection.

In the present analysis, we have included:

1. *13 twin women fulfilling lifetime DSM IV criteria for BN*, including 7 women from discordant twin pairs (1 MZ pair and 6 DZ pairs) and 6 women from concordant twin pairs (2 MZ and 1 DZ).

2. *25 healthy twin women*, including 6 healthy twin sisters of the women with BN (1 MZ and 5 DZ) and 18 women from 10 (5 MZ and 5 DZ female-female) healthy twin pairs.

All the study subjects were free from psychotropic medication at the time of the study. 4 women with BN had previously been on selective serotonin reuptake inhibitor (SSRI) medication, but had been drug-free for more than 1 year before the study.

The zygosity of all twin pairs was confirmed by genetic blood marker studies using the highly polymorphic, multiple genetic marker set used in the Paternity testing laboratory, National Public Health Institute, Helsinki, Finland.

After complete description of the study procedures to the subjects, written informed consent according to the Declaration of Helsinki was obtained from all participants. The Ethics Committees of Kuopio University Hospital and Helsinki University Central Hospital approved the study.

### Radiopharmaceutical

The radioligand [^123^I]ADAM [22] (MAP Medical Technologies Oy, Tikkakoski, Finland) was used for imaging of brain serotonin transporters. Thirty minutes before its intravenous injection, the subjects were given 400 mg of potassium perchlorate orally to reduce [^123^I]-uptake in the thyroid and the salivary glands. Injected radioactivity of [^123^I]ADAM varied between 139-231 MBq.

### SPET imaging and image reconstruction

The SPET scannings took place 5 hours after the injection of [^123^I]ADAM with a Philips Picker Prism3000XP three-headed gamma camera with ultra-high-resolution fan-beam collimators (Philips Medical Systems, Cleveland, OH, USA). The fan-beam focus of the collimator was 535 mm. We used 120 degree orbit in a stepwise mode and a symmetrical energy window (159 keV; 20% wide, 143 keV-175 keV). The radius of rotation varied between 130-160 mm, depending on the patient. The subjects' heads were positioned with a crossed laser beam system to the centre of rotation. Scans were acquired with a 128 × 128 matrix size using 120 projection angles (40 projections/detector). The acquisition time was 45 s per projection angle, resulting in an average of 20 kcts.

All reconstructions and image analyses were done on a HERMES software system (Hermes Medical Solutions, Stockholm, Sweden). The images were reconstructed iteratively (8 subsets with 6 iterations) using HOSEM (OS-EM V5.201 by R. Larkin) and transverse slices were reconstructed. Attenuation correction (Chang's first-order approximation) was performed using the linear attenuation correction (μ = 0.110 cm^-1^). Butterworth filter with cut-off frequency of 1.2 cm^-1 ^and order 15 was used for post-filtering of the images.

### Quantification of SERT availability

**Figure 1:**
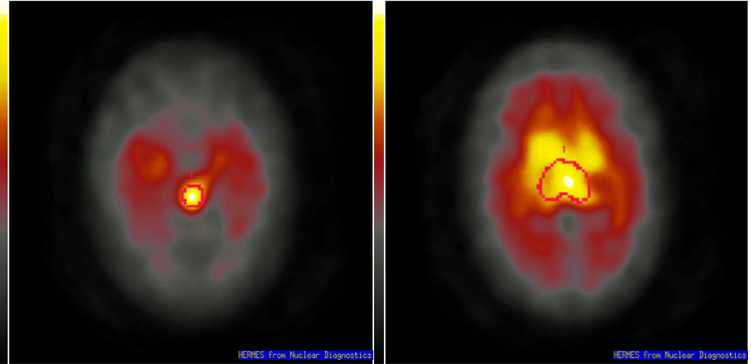
The [^123^I]ADAM template (mean image of scans of 15 healthy women) and the midbrain (left) and thalamus (right) volumes of interest.

We have previously created a brain template with a predefined volume of interest (VOI) map for [^123^I]ADAM images using BRASS (Brain Registration and Analysis of SPECT Studies) software (Hermes Medical Solutions, Stockholm, Sweden) on a HERMES workstation [27]. We used this template as a basis for automated registration and realignment of the scans done for the subjects in the study. The anatomically standardized (stereotactic) images were used for automated VOI quantification. SERT availability was assessed in VOIs of fixed size of the midbrain, the thalamus and the cerebellum, which was used as a reference region [34] (Figure 1.). We chose to restrict the data analyses to these regions on the basis of the previous data showing the greatest SERT binding and the least test-retest variability in the midbrain and the thalamus [23]. The voxel size was 2 × 2 × 2 mm and the slice separation 4 mm. Both the midbrain and the thalamus VOIs were placed on 2 consecutive slices and consisted alltogether of 94 (midbrain) and 386 (thalamus) voxels. The cerebellum VOI was placed on 3 consecutive slices and consisted of 1806 voxels. As the location of the midbrain activity varied a little between the subjects, more than the location of the thalamus activity, we chose to alter the position of the midbrain VOI manually, if it did not seem correct. This was done without altering the size of the VOI or the level (slice) on which the VOI was positioned by the automated procedure. All image reconstructions, registrations to the template and possible moving of the midbrain VOIs were performed by a nuclear medicine physician, who was blind to subject identity and diagnosis.

SERT binding was estimated by using the formula Specific Binding Ratio (SBR) = (mean counts in the target area – mean counts in cerebellum)/mean counts in cerebellum. This model has been shown to correlate well with the graphical analysis with arterial plasma input for [^123^I]ADAM-studies [35]. Half-life correction was done for calculated SBR values.

Non-specific uptake was quantified by dividing the mean counts/pixel of the cerebellar VOI by the injected activity.

### Statistical analysis

The data were analyzed using the Stata software (release 8.2; Stata Corp., College Station, TX, USA). If needed, the variables were log transformed to normalize their distribution.

In accordance with our study objective of examining the possible (genetic) differences of state and trait dependent SERT binding, we distinguished 3 groups of subjects: BN women, their healthy female co-twins and unrelated controls. State differences would be revealed by observed differences between BN women and their co-twins, whereas any differences observed between healthy co-twins and unrelated controls would be due to trait effects, given that the healthy co-twins share childhood experiences as well as at least half of their segregating genes with their affected co-twin. Our sample size did not permit us to do analyses by zygosity.

First, we compared all the twins as individuals. When twins are analyzed as individuals rather than as pairs, the observations and their error terms between members of a pair may be correlated. Therefore, to account for individuals nested within twin pairs, we used techniques derived from hierarchical linear modeling to adjust for clustering of twins within twin pairs. For comparison of the three groups, i.e. the diseased subjects, their healthy co-twins and the healthy control twin pairs we used one-way analysis of variance, and then appropriate two-group tests. Multiple linear regression analyses were used to test the effects of multiple explanatory variables on a response variable. In Stata, the procedures SVYMEAN, SVYTEST and SVYREG permit derivation of the proper standard errors, variances, confidence intervals and P values, correcting for dependency within a twin pair.

Second, we made within-pair comparisons to test the significance of intra-pair differences in SERT binding in twin pairs discordant for BN and in healthy twin pairs by using the paired nonparametric Wilcoxon test. Third, we compared the mean intrapair difference in SERT binding between twin pairs discordant for BN and healthy control twins by using the nonparametric Mann-Whitney U-test.

We also performed a *post hoc *analysis of the purging BN women against all the healthy women combined. This decision was based on the clinical observation that purging bulimia is often more severe in its course as compared to non-purging type bulimia. Further *post hoc *analyses were done on the effects of psychiatric co-morbidities on study results.

Data are shown as mean ± standard deviation. P values less than 0.05 were considered statistically significant.

## Results

### Demographic variables and behavioral assessments

The mean body mass indices (BMIs) and ages were similar for the 13 women with BN, their twin sisters and the healthy control twins (Table 1.) The mean age of onset of bulimia was 18.3 y (SD: 2.9 y, range: 13 – 21 y) and its mean duration was 6.5 y (SD: 4.4 y, range: 6 months – 14 y). Of the 2 subjects with lifetime BN, one had been asymptomatic for 3 and the other for 5 years. 9 of the 13 bulimic individuals suffered from the purging subtype of BN, and 4 were diagnosed with the non-purging subtype of BN. Compensatory behaviors other than vomiting included diuretics and Ipecac abuse, excessive exercise, and fasting. 5 individuals had a past history of anorexic symptoms, but had been free from anorectic symptoms for more than 6 months prior to our study.

**Table 1:** Study subjects' ages and BMIs

	**Bulimics (n = 13)**	**Twin-sisters (n = 6)**	**Controls (n = 18)**	P*
**BMI (kg/m^2^)**	22.9 ± 3.2	23.6 ± 3.2	22.2 ± 3.5	0.68
**Age (years)**	24.8 ± 1.7	24.8 ± 2.1	25.3 ± 1.7	0.81

*Significant differences between the groups were calculated using ANOVA: p-values were corrected for clustered sampling.

There was 1 case with present and 7 cases with past history of major depressive disorder (MDD) in the BN group. None of the healthy sisters or healthy control women suffered from present MDD. One healthy control with a past history of MDD was excluded from the study.

### SERT availability in volumes of interest

The specific binding ratios of [^123^I]ADAM to SERTs in the midbrain and the thalamus are shown in Table 2. SERT binding was similar for the groups of BN probands, their unaffected sisters and healthy control women in both the midbrain (p = 0.14) and the thalamus (p = 0.85). The unaffected sisters did not differ from the healthy controls in neither the midbrain (p = 0.66) nor the thalamus (p = 0.66), and therefore we combined these two groups of healthy women. No significant differences were found in comparison of the BN women and all the healthy women combined (Table 3.). Removal of the 2 currently unsymptomatic women from the group of probands did not affect these results (data not shown). In a *post hoc *analysis, the subgroup of purging bulimic women differed significantly in their SERT binding from the healthy women in the midbrain (p = 0.03) but not in the thalamus (p = 0.35) (Table 3.).

**Table 2:** SBRs for women with lifetime BN, their healthy co-twins and healthy control women

	**Women with lifetime BN (n = 13)**	**Healthy co-twins (n = 6)**	**Healthy control women (n = 18)**	P*
**Midbrain**	2.23 ± 0.22	2.02 ± 0.29	2.07 ± 0.31	0.21
**Thalamus**	1.45 ± 0.23	1.38 ± 0.25	1.43 ± 0.29	0.86

*ANOVA, correction for dependency within twin pairs

**Table 3:** SBRs: Comparison of all healthy women with all BN women and the subgroup of purging BN women

	**Women with lifetime BN (n = 13)**	**All healthy women (n = 24)**	**Women with purging BN (n = 9)**	P*	P**
**Midbrain**	2.23 ± 0.22	2.06 ± 0.30	2.26 ± 0.19	0.08	0.03
**Thalamus**	1.45 ± 0.23	1.42 ± 0.28	1.50 ± 0.25	0.73	0.45

* Women with lifetime BN vs. all healthy women. t-test, correction for dependency within twin pairs

** Women with lifetime purging BN vs. all healthy subjects. t-test, correction for dependency within twin pairs

In further *post hoc *analyses, we investigated the effect of MDD and past history of AN on the results. SBRs in the midbrain and the thalamus were similar for the subjects with (n = 7) and without (n = 30) a history of MDD (in the midbrain: SBR_MDD _2.20 ± 0.22, SBR_no-MDD _2.10 ± 0.30, p = 0.38, and in the thalamus: SBR_MDD _1.48 ± .28, SBR_no-MDD _1.42 ± .26, p = 0.63). Regarding past AN, the mean SBRs for SERT binding were very similar for the BN women with (n = 5) and without (n = 8) past history of AN (in the midbrain: SBR_AN _2.25 ± 0.26, SBR_no-AN _2.21 ± 0.22, and in the thalamus: SBR_AN _1.50 ± 0.28, SBR_no-AN _1.42 ± 0.21). In multiple linear regression analysis, history of MDD or AN had no effect on the results, whereas the effect of purging remained significant (Table 4).

**Table 4:** Multiple regression analysis examining the relationship of SERT binding with purging BN and past histories of Major Depression and Anorexia Nervosa

**MIDBRAIN: R^2 ^= 0.10, F(3,17) = 3.92, p = 0.03**	**β**	**S.E. of β**	**p-value**
**Purging BN**	-.22	.09	.02
**History of major depression**	.08	.10	0.44
**History of AN**	-.08	.09	0.38
			
**THALAMUS: R^2 ^= 0.03, F(3,17) = 1.02, p = 0.41**	**β**	**S.E. of β**	**p-value**

**Purging BN**	-.10	.06	0.14
**History of major depression**	.02	.09	0.80
**History of AN**	-.04	.12	0.72

Correction for dependency between twin pairs done for

In within-pair comparisons, there were no differences in SBRs between a twin and her co-twin in either of the brain regions in neither the group of twins discordant for BN (n = 6 pairs) nor the healthy twin pairs (n = 9 pairs). The means of intrapair differences for SBRs (SBR of twin 1-SBR of twin 2 in a given area) were also similar for the discordant twin pairs and the healthy control twin pairs in both the midbrain and the thalamus regions. Within-pair comparisons were not done for the pairs discordant for purging lifetime BN as we had only two such pairs.

SERT binding in the cerebellum (i.e., non-specific uptake) did not differ between the groups (98.99 ± 17.53 counts/pixel/kBq for the subjects with lifetime BN, 93.38 ± 21.42 counts/pixel/kBq for the healthy co-twins and 96.52 ± 15.26 counts/pixel/kBq for the healthy controls, p = 0.73).

## Discussion

In this study, we examined the binding of a SERT-specific radioligand [^123^I]ADAM to the midbrain and the thalamus of women with bulimia nervosa, their healthy female co-twins and healthy controls. Against our initial hypothesis, we found no differences in SERT binding between the three study groups. However, in a *post hoc *analysis we observed increased SERT binding in the midbrain in the purging BN women. Our work extends the previous literature on the role of serotonergic pathways in the etiology of BN[36] and suggest differences in SERT function between the purging and the non-purging BN women. Our findings do not support the existence of a bulimia-related endophenotype.

The involvement of serotonergic system in BN has been suggested in a number of studies. Examples include blunted plasma hormonal response to drugs with 5-HT activity [5], worsening of symptoms after dietary depletion of tryptophan (precursor of 5-HT) [10], the beneficial effect of antidepressant medications in BN [12,13], and differences found in brain PET- and SPET-studies in SERTs [18] and 5-HT_1A_-receptors [20]. Furthermore, some disturbances of serotonergic system, such as vulnerability to the effects of tryptophan depletion [11] and elevation of cerebrospinal fluid concentration of 5-HT metabolite 5-hydroxyindolacetic acid (5-HIAA) [15], seem to persist after recovery. Alterations after recovery have also been found in the imaging studies of 5HT_2A_-receptors [16].

There are several possible explanations to our failure to find differences of SERT binding between the whole group of bulimics and the healthy subjects. First, there may be alterations of SERT function in other brain regions than were investigated in our study. Some existing data from functional magnetic resonance imaging studies suggest alterations in the lateral fusiform gyrus, the inferior parietal cortex, and the lateral prefrontal cortex [37]. PET and SPET studies with 5-HT_1A _and 5-HT_2A _ligands have found changes in the the frontal, parietal and cingulate areas [36]. As the binding of [^123^I]ADAM is scant and less reliable in these regions [23], we did not investigate them in our study. Second, the disturbances of serotonergic function in BN may not be found in SERTs, but instead in other parts of the serotonergic system. Two previous studies have suggested alterations in 5-HT_2A _and 5-HT_1A_-receptors [20,21]. Even though the SSRIs have been shown to be useful in the treatment of BN [38], it does not necessarily indicate a disturbance of SERT function in BN, as the mechanism of action of the SSRIs is not believed to be direct effect on SERT or increased synaptic 5-HT [39]. Desensitization of somatodendritic 5-HT_1A _autoreceptors in the midbrain raphe nucleus is one suggested mechanism; this would increase 5-HT in critical brain regions and at key receptor subtypes [40-43]. Third, there is also a possibility of impairments outside but in connection to the serotonergic system. According to one theory, 5-HT acts as a modulator in the homeostasis between dopamine, noradrenalin and GABA. Serotonergic drugs might help reinstate the homeostasis of this system [44]. Another suggested mechanism of action of the SSRIs is the potentiation of neurogenesis [45].

There is also the possibility that the inclusion of the two currently unsymptomatic BN women diluted the differences between the probands and the healthy women. Their inclusion was based on our initial hypothesis of a bulimia-related endophenotype. As we later observed, our results on the unaffected co-twins do not support the idea of a SERT associated endophenotype for BN. However, removal of these two women from the probands did not change our findings.

It is also possible that BN may in fact include biologically distinct subgroups. Purging bulimics have been found to differ from non-purging ones in certain personality variables (e.g. lower self-directedness, organization, personal standards, and higher novelty seeking) [46]. Our results point towards a difference of SERT function between these two subgroups, given that only the subjects with a purging-subtype of BN differed significantly from the healthy subjects in the midbrain SBRs. As this is a finding of a *post hoc *analysis and made in a small study sample, it needs to be confirmed in other studies.

There are some important differences between the present study and the previous study on SERT binding in BN [18]. The radioligand previously used was [^123^I]β-CIT, which binds to DATs and NETs as well as to SERTs [47-49], leaving a possibility that its binding to NETs in the thalamus has affected the findings. Another difference lies at the study groups. It is possible that our BN cases, identified from a population-based twin cohort, were different to the clinic-based cases in the previous study. Thus, treatment seeking characteristics, disease severity and other differences between the study groups may to a point explain the differences between these two studies. Also, the exclusion of cases with psychotropic medication from our study probably shifts the balance towards clinically less complex cases.

In our study, SERT binding was similar in the healthy co-twins of the bulimics and the other healthy women. It is not known whether the previously reported alterations of 5-HT function after recovery [11,15,16] represent "scarring" caused by BN or a disturbance of 5-HT function that is present before the actual symptoms, predisposing a subject for BN. The reported premorbid anxiety symptoms [14] suggest presence of vulnerability for BN and the evidence from family and twin studies suggests a genetic contribution to etiology of eating disorders [17]. If such a genetic trait (i.e., endophenotype) exists in SERT function, it should be evident in the unaffected co-twins of the BN probands. Our finding of similarity of SERT binding between the co-twins and the other healthy women is against our initial hypothesis and does not support the existence of a SERT-related trait for BN. However, the small number of the unaffected co-twins reduces the reliability of this conclusion and also prevented analyses separately by zygosity. The number of unaffected co-twins of the purging bulimics (n = 2) was even smaller: therefore, we cannot make assumptions on whether the observed increase in SERT binding in the purging BN women is a state- or trait-related increase in SERT binding.

Our results differed from the initial hypothesis also in the direction of the difference in SERT binding. Our initial hypothesis was reduced SERT binding in BN: however, what we found was increased SERT binding in the purging probands. Many studies suggest reduced 5-HT transmission in BN [4-10,16,18]. Also in many (but not all) SPET and PET studies, reduced SERT binding has been interpreted to indicate reduced 5-HT function. However, this assumption may not necessarily be true. Theoretically, reduced intrasynaptic 5-HT could lead to increased binding of the radioligand due to 1) less competition for the binding sites or 2) compensatory increase in the amount of SERT on the nerve terminals. Alternatively, reduced 5-HT could reduce SERT binding if the amount of SERTs on nerve terminals is reduced due to increased internalization of SERTs [50]. Recent PET studies with the SERT ligand ^11^C-DASB have assessed the effects of artificial alterations of intrasynaptic 5-HT. The findings to date are inconsistent, with reports of either decreased [51] or unchanged [52] SERT binding after decrease in intrasynaptic 5-HT or decreased binding after increase in intrasynaptic 5-HT [53]. The true nature of the relationship of intrasynaptic 5-HT concentration and SERT binding is thus yet to be discovered. Furthermore, not only the amount of intrasynaptic 5-HT but also disease specific alterations in number and affinity of SERTs can affect SERT binding. Therefore, we don't really know the true interpretation of our findings: we can only state that we found a difference in a direction opposite from our original hypothesis.

There are some limitations to our study. Our sample size is smaller than we initially aimed for. Even though we screened thousands of twins, the number of BN probands was smaller than we expected. The presence of three concordant pairs also reduced the number of the unaffected co-twins, limiting our ability to make conclusions on the endophenotype concept. One factor that clearly reduced the number of our cases was the exclusion of subjects with antidepressant medication. We acknowledge the limited power of our study and the considerable probalility of both type one (due to multiple comparisons) and type two (due to small sample size) errors. Furthermore, the inclusion of subjects with past histories of MDD and AN are possible confounds. These conditions often co-exist and exclusion of probands with past history of MDD or AN would have made our study sample even smaller. However, in post hoc analyses, neither of these conditions had effect on our results.

We did not control for phase of menstrual cycle, which has been implicated as source of variation in SERT binding [54]. However, a recent study with [^123^I]-β-CIT found no such effect [55]. On the other hand, we could fully exclude variation due to sex and age by including only females within a narrow age-range.

The radiotracer [^123^I]ADAM might have some properties that affect its usefulness in SERT imaging. The reported intra-subject test-retest variability of [^123^I]ADAM binding is 13% in midbrain and 16% in thalamus, which is, however, of the same magnitude as for other SERT SPET ligands [23]. There is also a report on lipophilic metabolites of [^123^I]ADAM [35], which, if present, might also affect our findings. We also can not exclude the possibility of different metabolism of [^123^I]ADAM between patients and healthy subjects as we did not do blood screening of its metabolism. The cerebellar activity, which mostly represents non-spesific binding of [^123^I]ADAM, did not differ between the groups.

Our template based realignment and fitting procedure is objective and repeatable, but may lead to some small errors of VOI placement and also dilute or emphasize some effects. However, this method was most suitable for our study as Magnetic Resonance Imaging (MRI) – based VOI definition was prevented by some of our subjects having contraindications for MRI. We tried to minimize the VOI placement errors by manually moving the midbrainVOI of fixed size within the fixed brain level, when necessary.

In summary, our results do not support the initial assumption of decreased SERT binding in BN or the existence of a SERT-related endophenotype for BN. Instead, the increased SERT binding in the midbrain of purging bulimics observed in this study suggests that the involvement of SERT function in this type of BN is stronger than in non-purging BN and differs from healthy subjects. However, due to small sample size and confounding factors our results need to be taken with caution and verified in larger samples of clinical BN cases.

## Competing interests

The author(s) declare that they have no competing interests.

## Authors' contributions

AK carried out and analysed the SPET studies, performed statistical analyses and was the main author of the manuscript.

AK-R and ES participated by planning the study design, in selecting the study subjects, and doing the psychiatric assessments, and by commenting on, revising and approving the manuscript.

TK participated by planning the SPET protocol and quantification of the SPET studies, commenting on, revising and approving the manuscript.

JK participated by collecting the Finnish Twin Cohort data, in planning of the BN study, and by commenting on, revising and approving the manuscript.

AA participated by planning the SPET protocol, commenting on, revising and approving the manuscript.

AR participated by planning the study design, commenting on, revising and approving the manuscript.

All the authors have read and approved the final manuscript.

## Pre-publication history

The pre-publication history for this paper can be accessed here:


